# Total Knee Arthroplasty in Ochronosis: A Rare Condition

**DOI:** 10.7759/cureus.33523

**Published:** 2023-01-08

**Authors:** Anil Arora, Getnet Asnake, Karan Pandav

**Affiliations:** 1 Orthopedics and Joint Replacement, Max Super Speciality Hospital, Patparganj, Delhi, IND

**Keywords:** ochronosis, total knee arthroplasty, alkaptonuria, black articular cartilage, homogentisic acid

## Abstract

Ochronosis is a rare metabolic disorder characterized by homogentisic acid deposition in the large joints and spine, resulting in progressive degeneration. We present two cases of ochronotic arthritis of the knee subjected to cemented total knee arthroplasty (TKA). These cases were diagnosed intraoperatively and later confirmed with a histopathologic examination. Orthopedic surgeons should be aware of the condition as the intraoperative finding of darkened cartilage might surprise them. After a five-year TKA follow-up, both of our cases showed better mobility and function.

## Introduction

Ochronosis is a rare metabolic disorder with a prevalence of one in 250,000-1,000,000 births [1]. This metabolic disorder occurs in the catabolic pathway of tyrosine and is caused by a deficiency of the hepatic enzyme homogentisate oxidase, leading to the accumulation of homogentisic acid in connective tissues [1]. The deposition of homogentisic acid leads to dark pigmentation, inflammation, and, later on, the degeneration of the tissue [1,2]. The deposition is seen on articular cartilage, bones, tendons, ears, sclera, skin, and aortic valves [3]. The accumulation of homogentisic acid in the bigger joints over the years results in articular cartilage degeneration and ochronotic arthritis. Commonly affected joints include the spine, hip, knee, shoulder, and sacroiliac [3]. Total knee arthroplasty (TKA) is indicated for patients with advanced ochronotic arthropathy of the knee [4,5].

The main aim of this paper is to increase awareness of this condition among orthopedic surgeons by presenting two cases of ochronotic arthropathy. In both cases, this condition was not suspected preoperatively and was diagnosed intraoperatively. This might come as an intraoperative surprise and pose a diagnostic dilemma to an unwary surgeon.

## Case presentation

Case one

**Figure 1:**
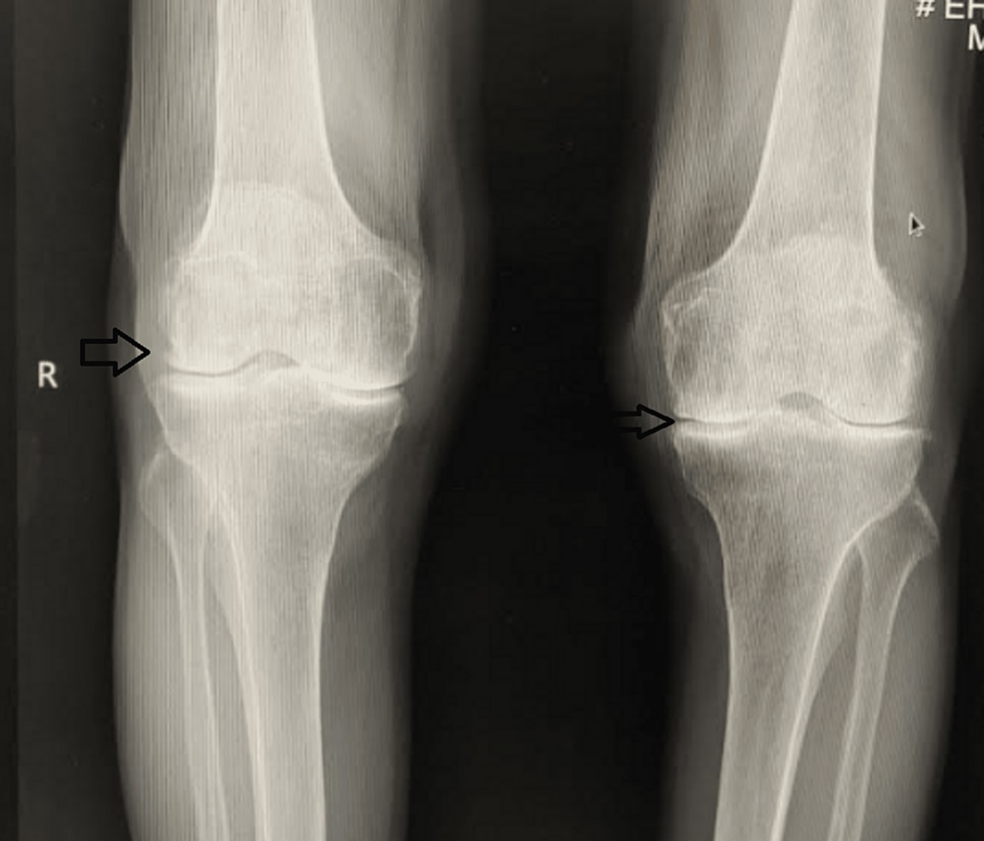
Anteroposterior view plain radiography of both knee joints suggesting features of arthritis (joint space narrowing and osteophytes).

**Figure 2:**
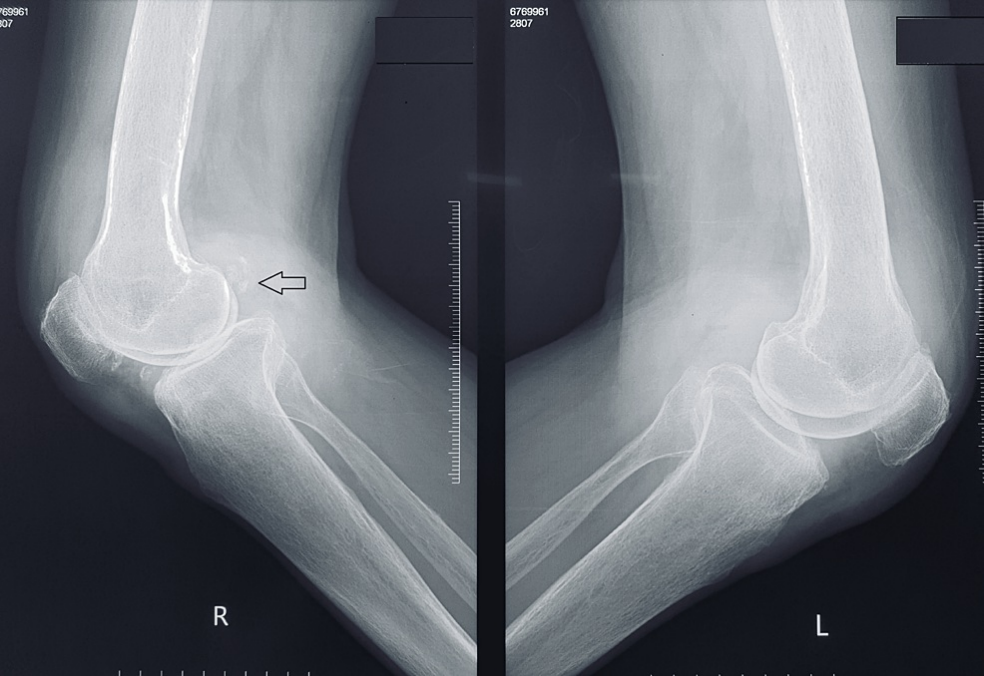
Lateral view plain radiography of both knee joints suggesting features of arthritis.

**Figure 3:**
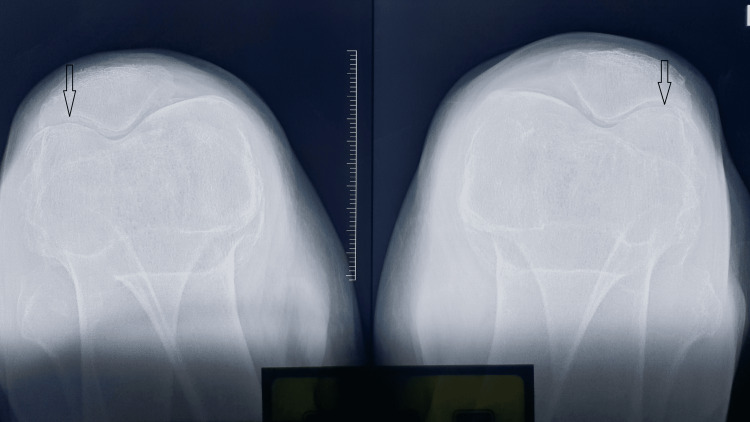
Skyline view plain radiography of both knee joints suggesting features of arthritis (patellofemoral joint space narrowing).

**Figure 4:**
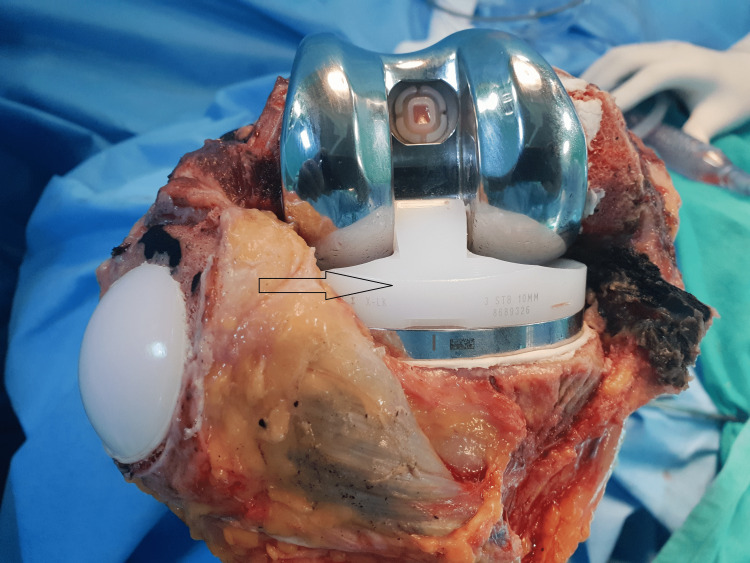
Total knee replacement implants positioned.

**Figure 5:**
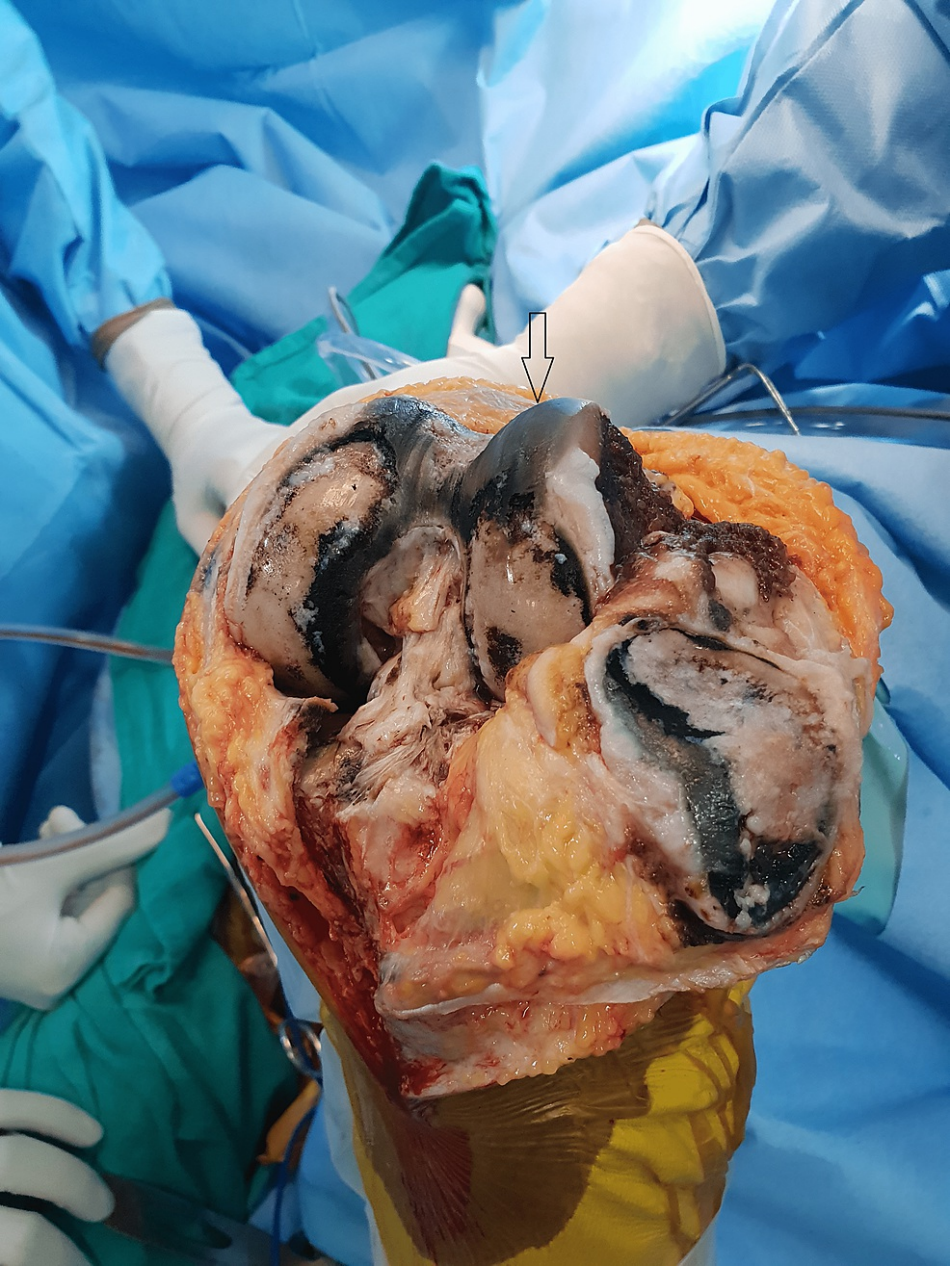
Dark pigmentation of articular cartilage and soft tissues.

**Figure 6:**
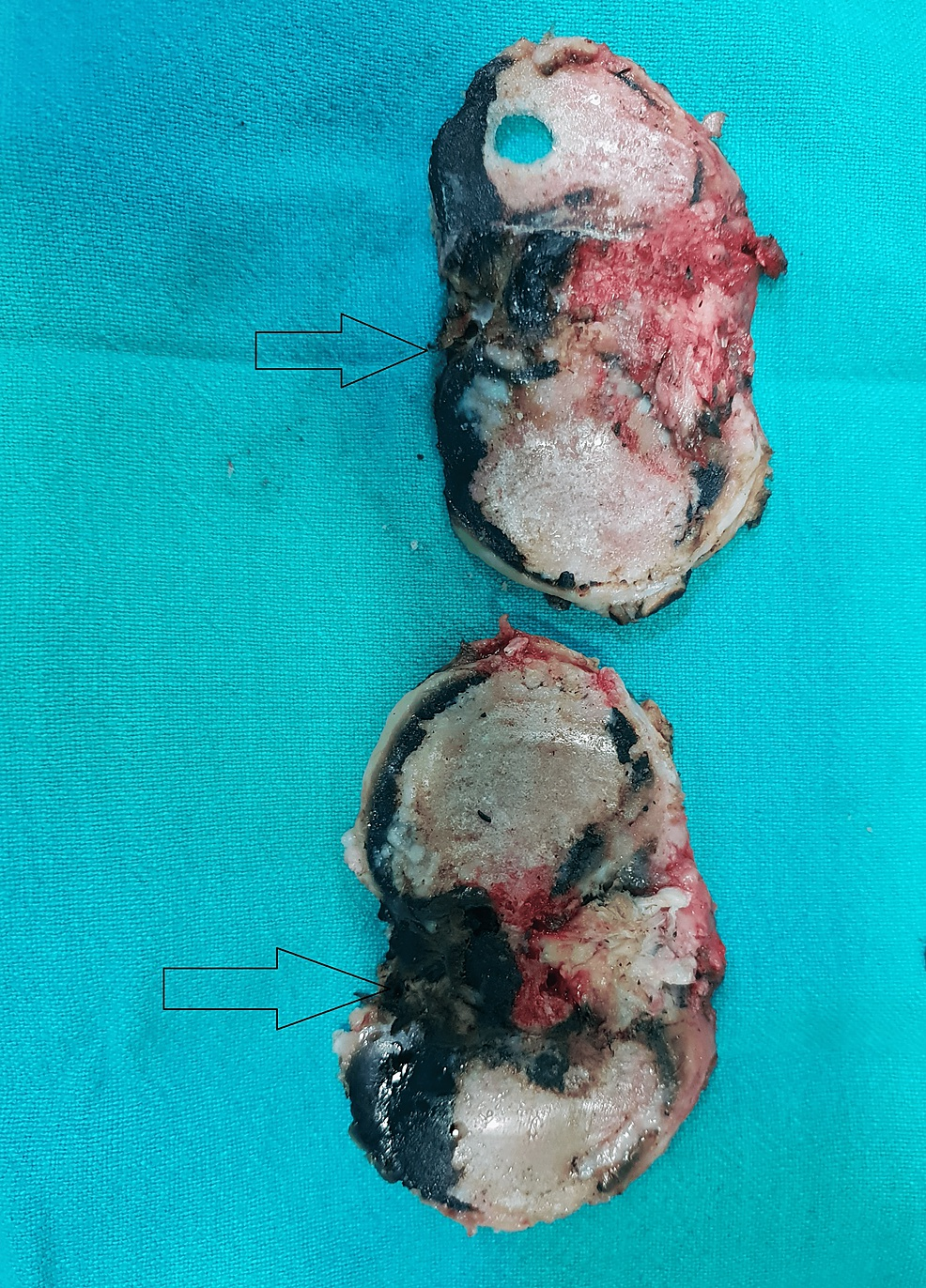
Darkened resected articular surfaces.

**Figure 7:**
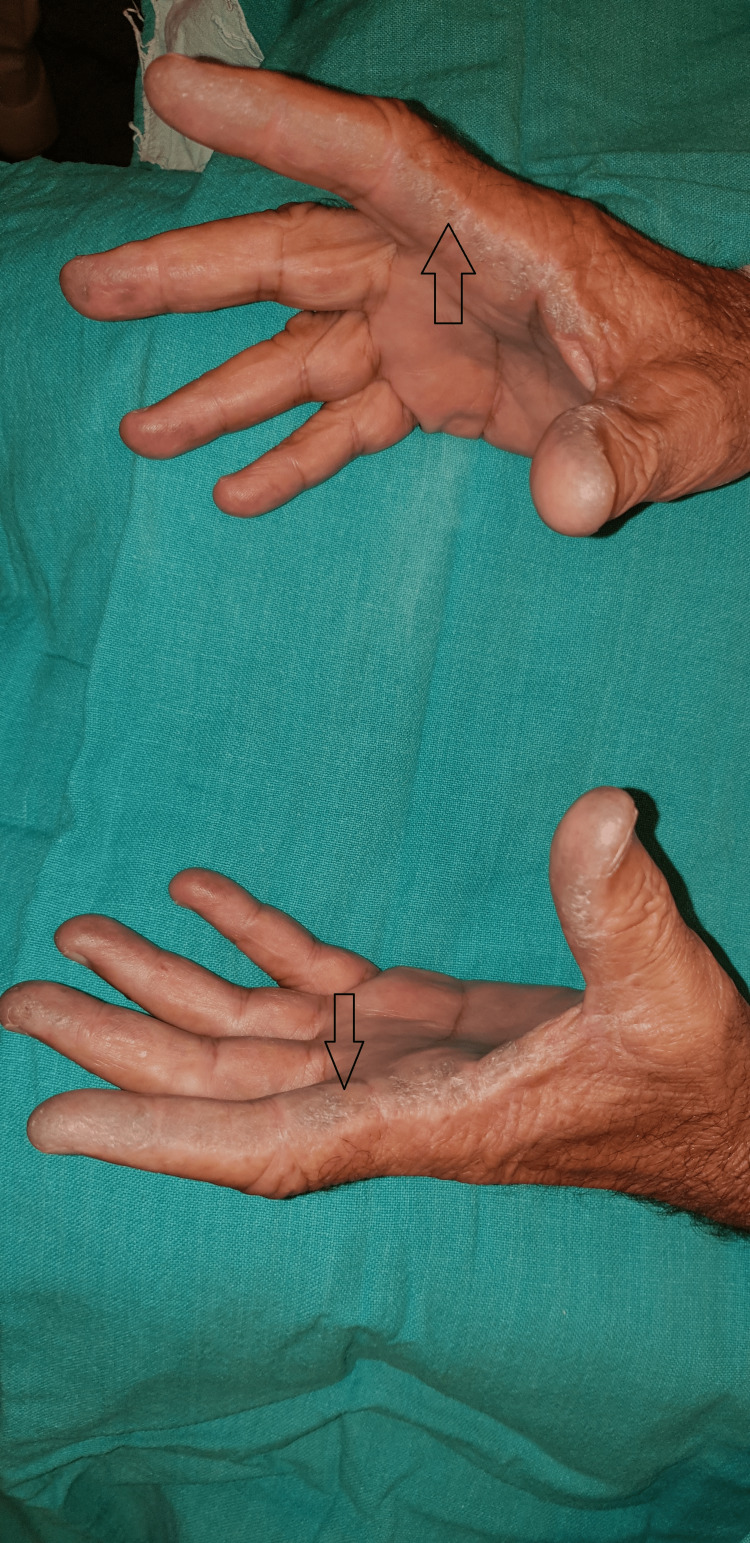
Darkened palmar skin over the radial aspect of right and left indexes.

**Figure 8:**
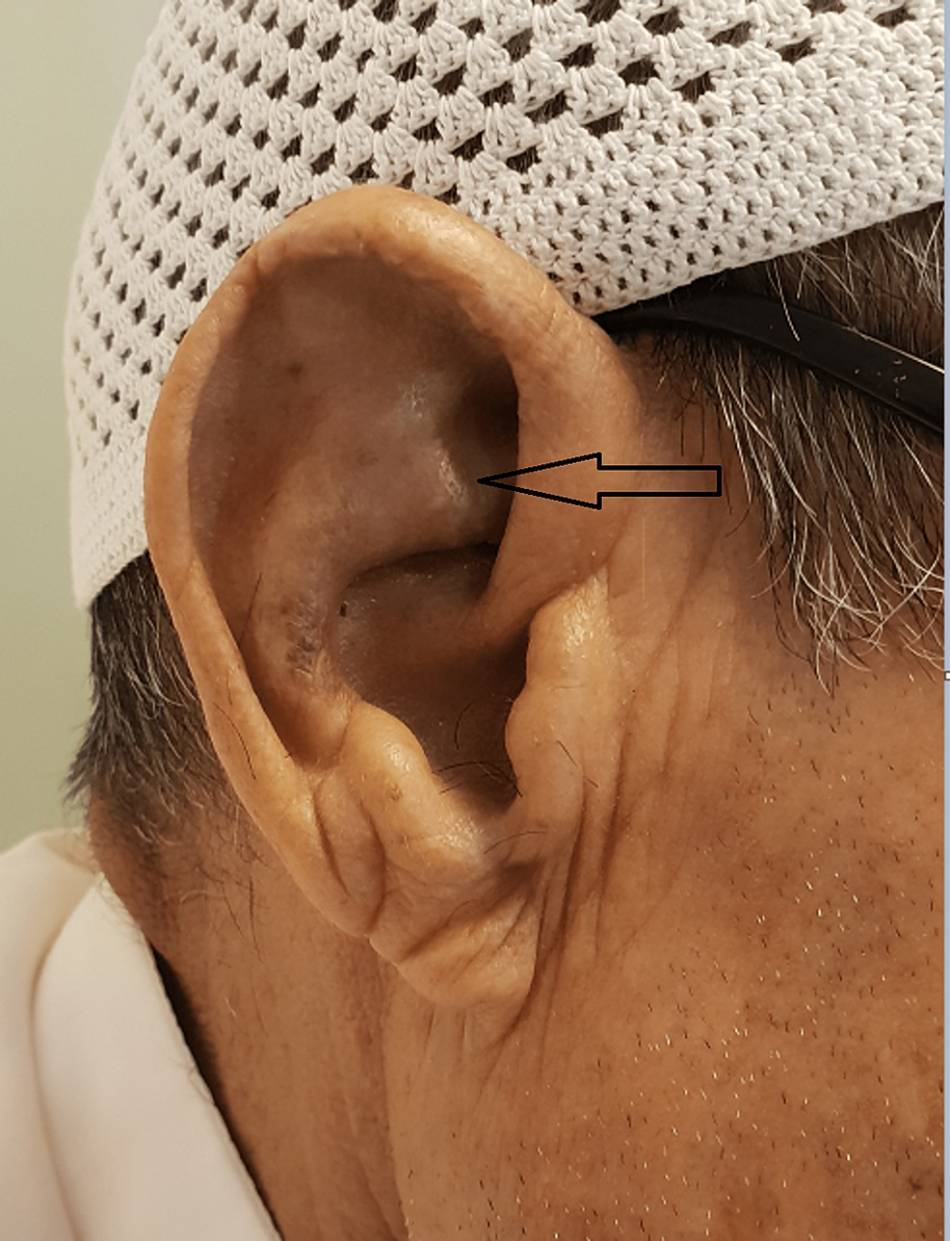
Darkened skin over the antihelix of the right ear.

**Figure 9:**
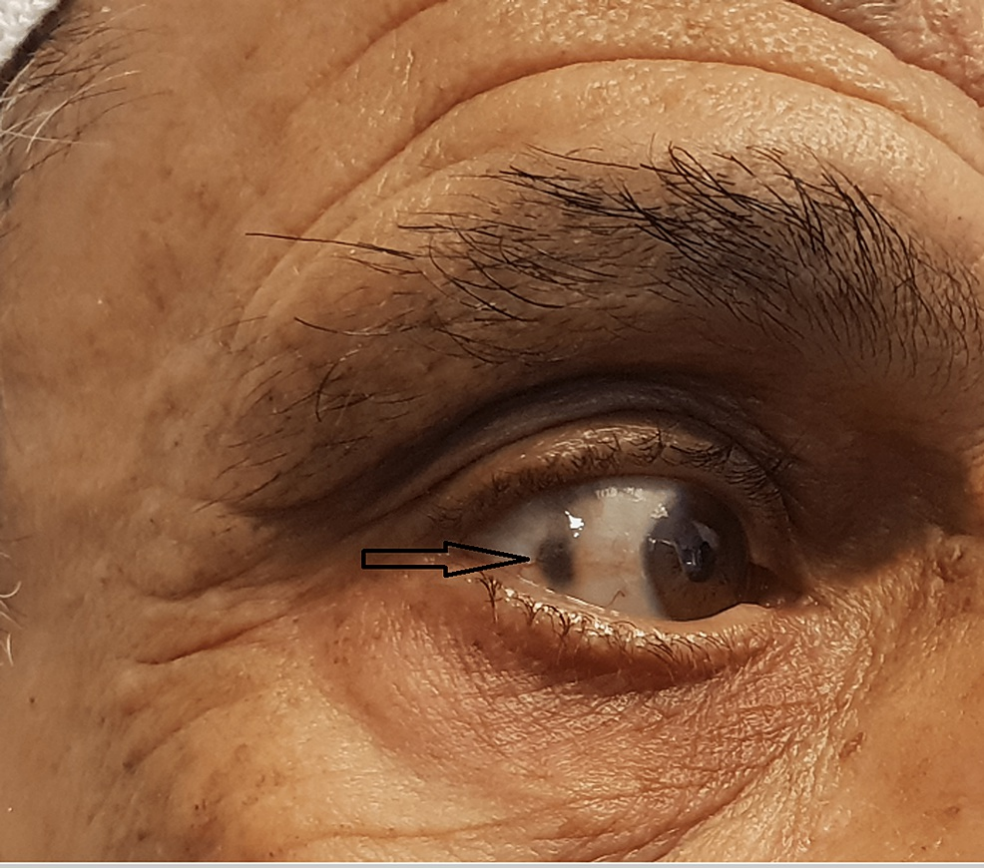
Darkened spot over the right sclera.

**Figure 10:**
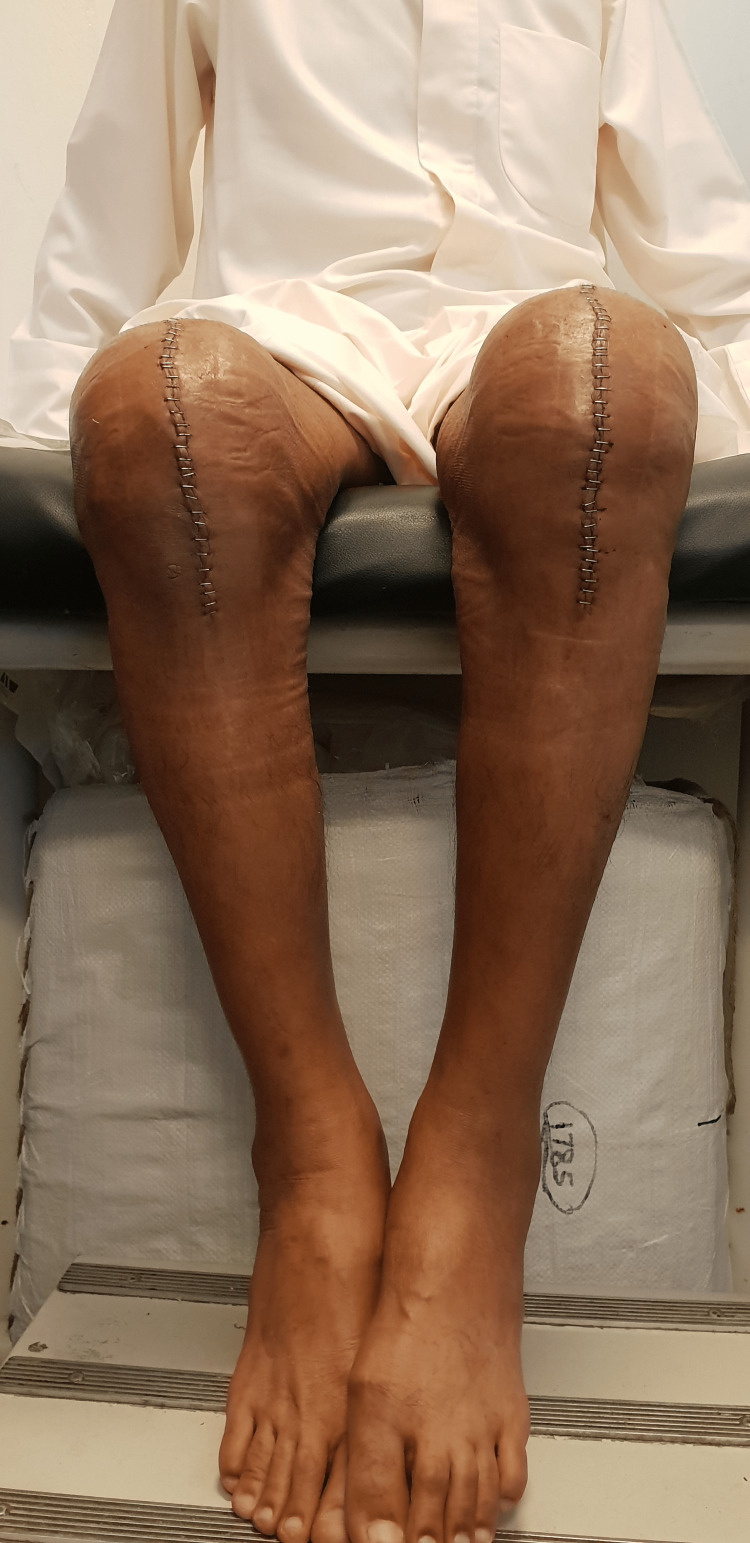
Postoperative knee flexion after two weeks.

An Iraqi man in his mid-60s came to us with five years of bilateral knee pain. A physical examination of both knees revealed synovial thickening, diffuse joint tenderness, and 15 degrees of varus deformity on both sides. The knee had a 0 to 120-degree range of motion. On plain radiographs of the knees, both knees had varus deformity and Kellgren-Lawrence grade IV arthritis [6] (Figures 1-3). The patient was taken for bilateral TKA. Articular cartilages of the patella, femoral condyles, and tibial condyles were found to be degenerating and darkened during surgery. The medial and lateral menisci were also dark (Figures 4, 5). Using Depuy Synthes (Warsaw, USA) PFC Sigma Implants, cemented posterior stabilized TKA was performed (Figure 6). We did not suspect the diagnosis of ochronosis preoperatively. In the postoperative period, we looked for further relevant physical examination findings suggestive of ochronosis. The examination revealed darkly pigmented lesions over the right outer ear, sclera, and radial aspect of both index fingers that indicated ochronosis (Figures 7-9). After surgery, plain radiographs of both knees revealed satisfactory alignment and implant placement. The range of motion in both knees was 0 to 90 degrees two weeks after surgery (Figure 10). The patient’s range of motion in both knees was 0 to 110 degrees without any pain at the five-year follow-up.

Case two

**Figure 11:**
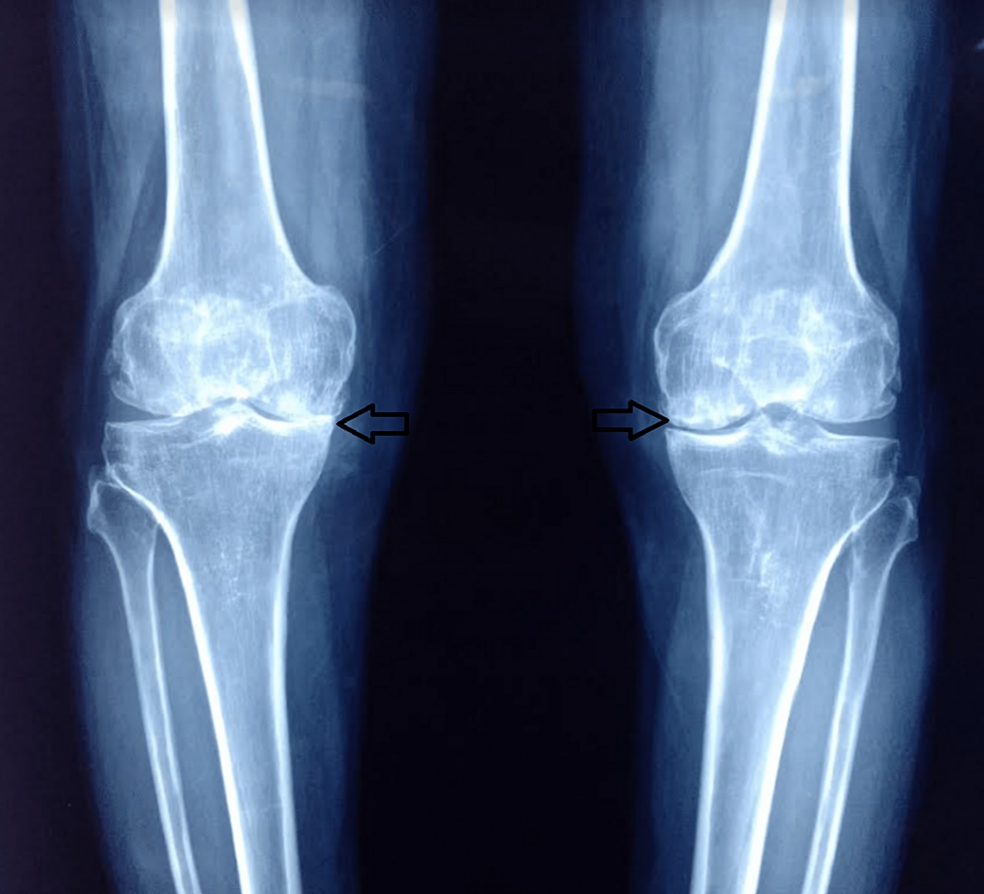
Preoperative anteroposterior plain radiography of both knee joints suggesting features of arthritis.

**Figure 12:**
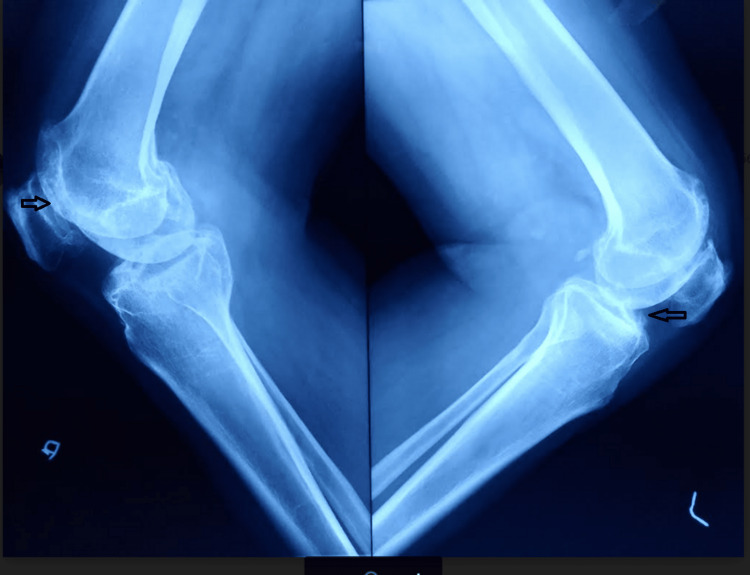
Preoperative lateral view plain radiography of both knee joints suggesting features of arthritis.

**Figure 13:**
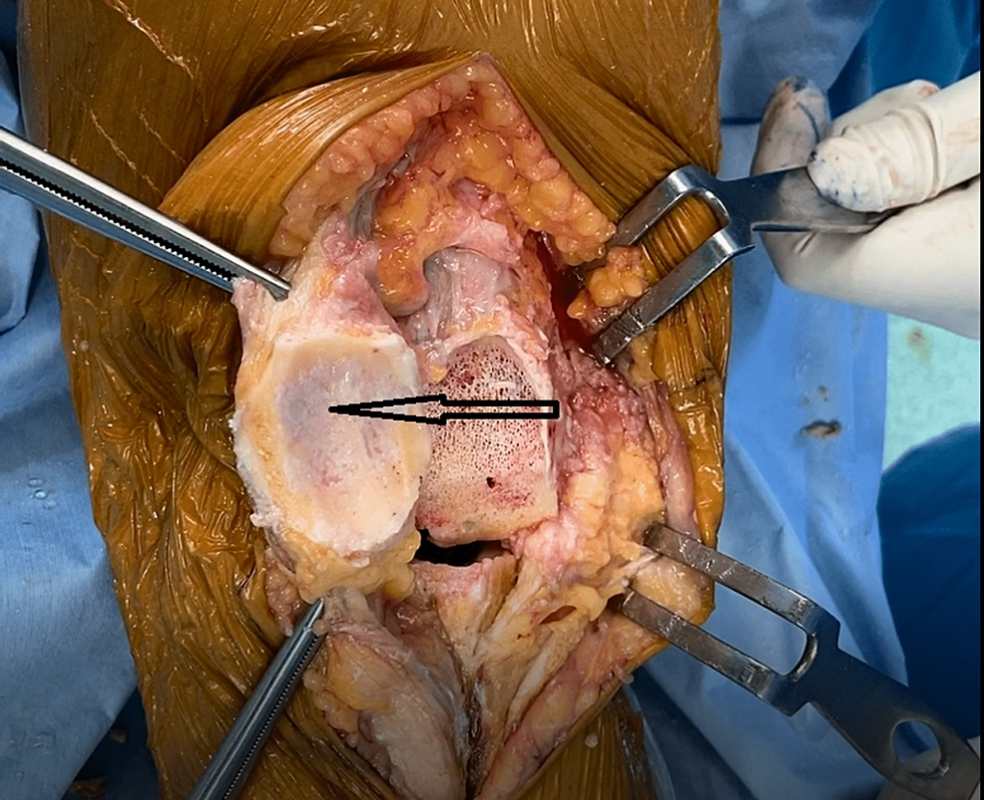
Pigmented patellar articular cartilage.

**Figure 14:**
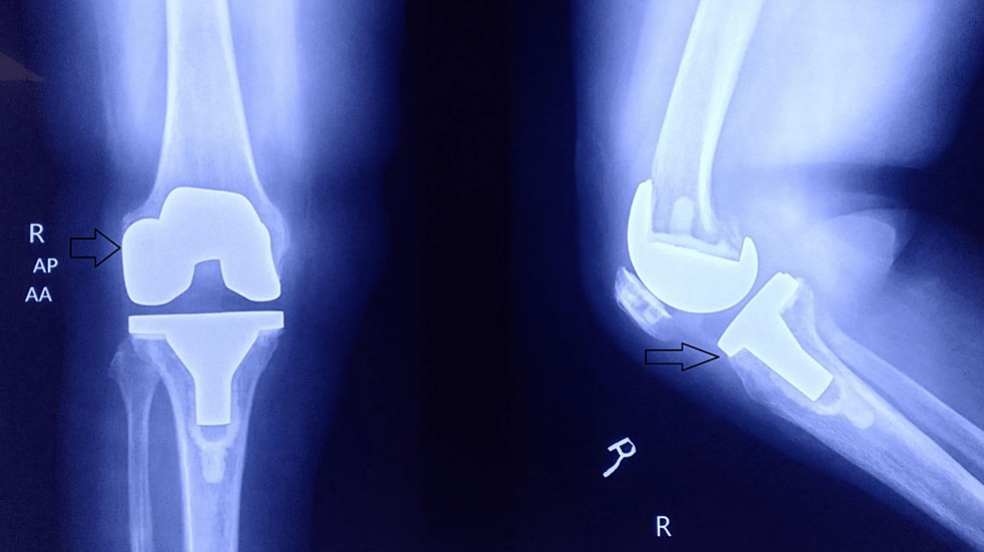
Postoperative radiograph of the right knee joint with good alignment and implant position.

**Figure 15:**
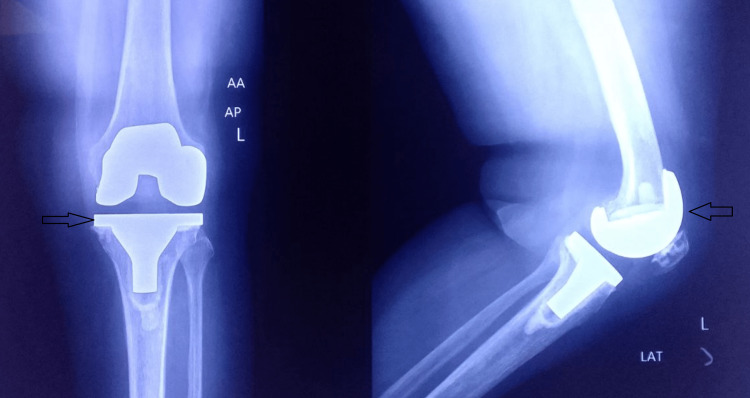
Postoperative radiograph of the left knee with good alignment and implant position.

A 60-year-old Indian female presented with significant progressive bilateral knee pain, limited ambulation, and difficulty ascending stairs. She had persistent synovitis in both knees, and her previous doctors had diagnosed her with seronegative rheumatoid arthritis. On blood examination, her rheumatoid factor (RF) and anti-cyclic citrullinated peptide (anti-CCP) antibodies were negative. She was put on oral methotrexate and oral hydroxychloroquine, disease-modifying anti-rheumatic drugs (DMARDs) by her previous doctors. Physical examination revealed an antalgic gait with a 5 to 110-degree (left knee) and 0 to 100-degree (right knee) range of motion. Plain radiography revealed grade IV arthritis with a varus-aligned knee (Figures 11, 12). She was taken in for bilateral TKA. Intraoperatively, we found darkened articular cartilages, menisci, and softened bone (Figure 13). Depuy Synthes (Warsaw, USA) PFC Sigma Implants were used in a cemented posterior stabilized TKA. A postoperative radiograph showed a well-aligned knee (Figures 14, 15). Fifty-four months of postoperative follow-up showed a satisfactory clinical outcome.

## Discussion

Ochronosis metabolic arthropathy is a rare degenerative joint disease characterized by the deposition of homogentisic acid on the articular cartilages of large joints and the spine. Symptomatic ochronosis in joints is usually seen after the fourth decade of life and clinically resembles osteoarthritis [1,7]. There is no sex predilection. Although the disease affects both sexes equally, males exhibit an early onset of arthritic symptoms, and females show a severe form of the disease [7]. From previously published literature, most diagnoses of knee ochronotic arthritis are made intraoperatively [4,7]. Similarly, in our cases, the diagnosis was made intraoperatively, and other suggestive physical findings were noted retrospectively in the postoperative follow-up period. An intraoperative synovial biopsy sent for histopathologic examination revealed ochronosis. A systematic review of previous publications suggested that no particular implant design proved to be superior [4]. In our cases, the quality of the posterior cruciate ligament was questionable, and we preferred a posterior cruciate ligament-sacrificing implant in both cases. In a critical review of 13 case reports, no difference was suggested between cementless and cemented total knee replacement for ochronotic arthritis [8]. In our cases, the bone was extremely soft, and we preferred a cemented total knee replacement. The deposition of homogentisic acid in the patellar articular cartilage and tendon was thought to cause chemical irritation and tendon weakness [9]. We performed patella resurfacing in our cases because the patellar articular cartilage had dark pigment deposits. To prevent the avulsion of the ligamentum patellae during patella resurfacing, the patella was gently everted with great care. The excision of hypertrophied synovium has resulted in massive intraoperative and postoperative bleeding in earlier cases described in the literature [10]. In our cases, we used a tourniquet and administered tranexamic acid preoperatively to prevent bleeding. As a result, intraoperative and postoperative bleeding was minimal in both of our cases.

In the literature, as no specific postoperative rehabilitation guideline has been mentioned, we used a similar protocol to that used for standard total knee replacement patients. Total knee replacement has a good outcome in patients who are not responding to conservative management of ochronotic knee arthritis [5,11]. In our cases, 54-month and 60-month postoperative follow-ups showed pain-free joints with a satisfactory range of motion.

Ochronotic arthropathy may have persistent synovitis before surgery [10]. Our second case had persistent synovitis with bilateral knee effusion preoperatively. Her inflammatory arthritis tests were negative, and she was not suspected of having ochronotic arthritides. She was diagnosed with seronegative rheumatoid disorder by her previous doctors. Her RF and anti-CCP antibodies were negative. She was put on oral methotrexate and oral hydroxychloroquine and DMARDs by her previous doctors. Awareness of ochronotic arthropathy in the orthopedic community will prevent these kinds of errors. Unaware surgeons may encounter an intraoperative surprise with darkened articular cartilage and soft tissues caused by ochronotic arthritis.

## Conclusions

Ochronosis is a rare metabolic disease affecting large joints and the spine with the hallmark of dark pigment deposition on articular cartilage, ligaments, and tendons. The diagnosis is usually delayed due to non-specific symptoms. Patients with ochronotic arthropathy might be misdiagnosed as seronegative rheumatoid disorders due to persistent synovitis. The diagnosis is usually made intraoperatively. Further studies are needed to establish the superiority of a particular implant design. TKA has a good outcome in terms of pain reduction and function with a five-year follow-up after surgery. Long-term follow-up is needed to establish the outcome of TKA in ochronotic arthropathy.

## References

[1] Harun M, Hayrettin Y, Serhat M, Cuneyt M, Fırat F, Ufuk O (2014). A rare cause of arthropathy: an ochronotic patient with black joints. Int J Surg Case Rep.

[2] Ulucay C, Ozler T, Altintas F, Inan M, Onur A, Kocadal AO (2013). Arthroplasty in ochronosis “tips and pearls in surgery”: case series. J Arthritis.

[3] Couto A, Sá Rodrigues A, Oliveira P, Seara M (2018). Ochronotic arthropathy-a rare clinical case. Oxf Med Case Reports.

[4] Lee WC, Tan TL, Chan YH (2019). Total knee arthroplasty in ochronosis arthropathy: a case report and systematic review. Case Rep Orthop.

[5] Karaoğlu S, Karaaslan F, Mermerkaya MU (2016). Long-term result of arthroplasty in the treatment of a case of ochronotic arthropathy. Acta Orthop Traumatol Turc.

[6] Kohn MD, Sassoon AA, Fernando ND (2016). Classifications in brief: Kellgren-Lawrence classification of osteoarthritis. Clin Orthop Relat Res.

[7] Demir S (2003). Alkaptonuric ochronosis: a case with multiple joint replacement arthroplasties. Clin Rheumatol.

[8] Ozmanevra R, Güran O, Karatosun V, Günal I (2013). Total knee arthroplasty in ochronosis: a case report and critical review of the literature. Eklem Hastalik Cerrahisi.

[9] Gil JA, Wawrzynski J, Waryasz GR (2016). Orthopedic manifestations of ochronosis: pathophysiology, presentation, diagnosis, and management. Am J Med.

[10] Patel VG (2015). Total knee arthroplasty in ochronosis. Arthroplast Today.

[11] Ranganath M, Saraf S, Naik RS, Ashok MS (2021). A study on total knee replacement in alkaptonuric patient. Int J Orthop Sci.

